# Pelvic congestion syndrome and May-Thurner syndrome as causes for chronic pelvic pain syndrome: neuropelveological diagnosis and corresponding therapeutic options

**DOI:** 10.52054/FVVO.13.2.019

**Published:** 2021-06-28

**Authors:** M Possover, S Khazali, A Fazel

**Affiliations:** Centre for Endometriosis and Neuropelveology, Possover International Medical Center, Zurich, Switzerland; Centre for Endometriosis and Minimally Invasive Gynaecology (CEMIG), Ashford and St. Peter’s Hospitals NHS Foundation Trust, Chertsey, United Kingdom; Service de Gynécologie Obstétrique, APHP-Hôpital, Lariboisière, 2 rue Ambroise Paré, Paris, France

**Keywords:** Neuropelveology, vascular entrapment, May-Thurner syndrome, pudendal pain, Coccygodynia, Vulvodynia

## Abstract

**Objective:**

To report on diagnosis and management of pelvic congestion including the May-Thurner syndrome (MTS) as potential etiologies for intractable pelvic neuropathic pain.

**Design:**

Retrospective study of women presented with intractable pelvic neuropathic pain, who had left sided venous uterine plexus above 6mm with reversed and slow flow on Doppler, with dilated arcuate veins passing through the uterine muscle. Those with suspicion of MTS underwent further radiological investigations and if applicable, endovascular interventions.

**Intervention:**

61 consecutive patients were included. 14 with visceral pain presumed to be caused by Pelvic Congestion Syndrome were treated by ovarian vein embolization. An improvement of pain was observed in all patients – mean pain reduction of 3.93 points, from 7.21 (±1.42; 4-10) to 3.28 pts (±1.54; 1-6) over 6 months (p<0.01). 47 presented with pelvic somatic neuropathic pain; 19 underwent endovascular intervention (angioplasty, stenting) and finally all of them a laparoscopic exploration/decompression of the sacral plexus and the endopelvic portion of the pudendal nerves, with an overall VAS reduction from 8.56 (±1.1712;7-10) to 2.63 (±1.53; 0-6) at one-year- follow-up (p<0.01).

**Conclusion:**

Laparoscopic exploration/decompression of the nerves seems to be effective in a carefully selected group of patients. Endovascular interventions for pelvic somatic neuropathies may not be an effective treatment. We recommend that Doppler studies of the uterine vessels are performed as an extension to gynaecological examination in women with intractable pelvic pain.

## Introduction

Pelvic varicose veins are very common in multiparous women, and are most often asymptomatic, but what seems to be underestimated is that pelvic varicose can be secondary to MTS or NCS and may lead to both visceral pain (pelvic congestion) and even somatic pain by compression and irritation of the sacral plexus and its endopelvic collaterals, causing pudendal and/or gluteal neuralgias and sacral radiculopathies (Lemasle et al, 2001). These medical conditions are mostly overlooked and under diagnosed as it sits on the edge of three medical specialties: neurology, gynecology and vascular medicine, but should be more in the focus of gynaecology, especially since the diagnosis can be suspected by gynaecologist performing transvaginal ultrasound.

The May–Thurner syndrome (MTS), also known as Cockett’s Syndrome or the iliac vein compression syndrome (Butros SR, Liu R et al, 2013) is a condition in which compression of the left iliac common vein by the right common iliac artery may cause discomfort, swelling, pain or deep vein thrombosis in the iliofemoral veins. MTS is often unrecognized; however, current estimates are that this condition is twice as common in women than in men (Sugimoto et al, 2001). The second well- known venous system compression on the left, the nutcracker syndrome (NCS), results most commonly from the compression of the left renal vein between the abdominal aorta and superior mesenteric artery, although other variants exist (Oteki et al, 2004). The compression causes renal vein hypertension, leading to hematuria (Barnes RW, Fleisher HL et al, 1988) and abdominal pain which may improve or worsen depending on positioning (White et al, 2017). Patients may also have orthostatic proteinuria (Lemasle et al, 2001) . It is important to consider these two syndromes in patients who have no other obvious reason for hypercoagulability and who present with left lower extremity thrombosis. The diagnosis could be confirmed by imaging techniques including magnetic resonance venography, venogram or intravascular ultrasound since the flattened vein may not be noticed on conventional venography.

In this article, we report on our neuropelveological experience with these two syndromes, especially the MTS.

## Methods

### 

**Figure 1:**
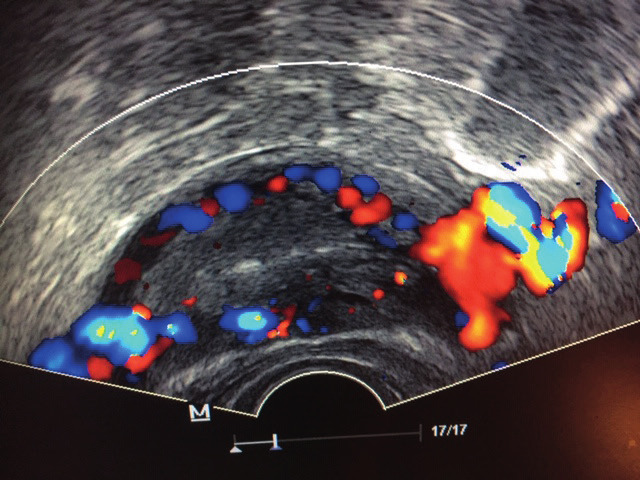
— Left sided dilated uterine vein >6mm in diameter with dilated arcuate veins passing through the uterine muscle.

This retrospective study, included all patients who presented at the Possover International Medical Center between 2014 and 03/2020 with chronic pelvic pain with dilated veins >6mm predominant on the left side (<4mm on the right) and dilated arcuate veins passing through the uterine muscle (Fig. 1). Patients with pelvic neuropathic pain (axonal lesion, for example postsurgical nerve injuries), pelvic nerve tumours (pelvic/genital carcinoma, schwannoma, teratoma…), infiltrating parametric endometriosis especially with involvement of the pelvic nerves and patients with postoperative nerve injuries (sutures, mesh…) were excluded from this study. All patients were refractory to a diverse range of medical treatments (NSAID, anticonvulsant medications, morphine, as well as to hormone-induced amenorrhea for at least 6 months.

**Figure 2:**
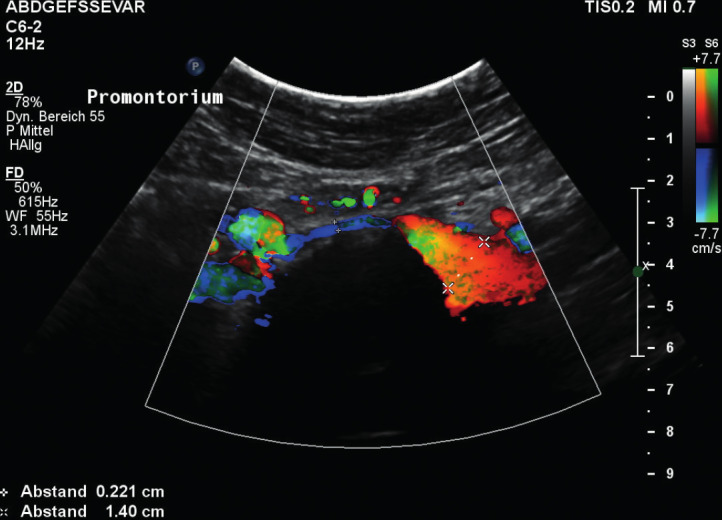
— Stenosis of the left common iliac vein.

In neuropelveological assessment for chronic pelvic pain of unknown etiology, clinical examination focuses on inspection of the internal/ external genital organs and the pelvic nerves (Possover and Forman, 2014) supported by urodynamic testing and vaginal ultrasonography, including duplex ultrasound investigation of the uterine and pelvic sidewall veins. Normal venous uterine plexus appears as straight tubular structures with a normal diameter <4mm. In patients with pelvic varicosities, an ultrasound typically shows dilated and tortuous veins with a diameter >6mm, with reversed and slow flow, that may be located on both sides of the uterus, or only unilaterally (García-Gimeno et al, 2009 - Lechter et al, 1991). In these selected patients, sonographic examination was extended by abdominal ultrasonography with pulsed color Doppler of:

- The ovarian veins at the anterior side of the psoas muscle (Possover , 2015). In MTS, ovarian reflux is left sided, spontaneous, permanent, and has little or no modulation by breathing.
- The iliac vessels: In a MTS, a stenosis of the left common iliac veins can be observed between the right common iliac artery and the 5th lumbar vertebra (Fig 2).

Patients were classified in two groups: “Visceral Pain” (by irritation of the inferior hypogastric plexus) and “Somatic Pain” (by irritation of the pelvic somatic nerves). Table 1 describes the clinical features and symptoms of these two types of pain.

**Table I:** Visceral Versus Somatic Pain: Symptoms (20).

Visceral pain	Somatic pain
**Pain quality:** Vague; poorly localized in the entire lower abdomen with radiation to the lower back; dull in nature	**Pain quality:** Allodynia; similar to an electrical shock; very specific location; precise and clear pain description; lack of vegetative symptoms
**+ Vegetative symptoms:** Malaise/oppression/syncope Fatigue Irritability Pupil dilation Salivation inhibition Tachycardia Nausea/vomiting Pallor Diaphoresis Anxiety	**+ Caudal radiation to the corresponding dermatome(s)**
	**+ Pelvic motor dysfunction:** Pelvic organ dysfunctions Sexual dysfunction Locomotion dysfunction

All included patients underwent a routine MRI FSE T2-weighted sequence with fat saturation (and/or 3D volumetric protocol) to evaluate dilated ovarian veins and possible stenosis of the iliac vein (Fig. 3). When MTS, NCS or pelvic congestion syndrome was suspected, retrograde venography was performed.

**Figure 3:**
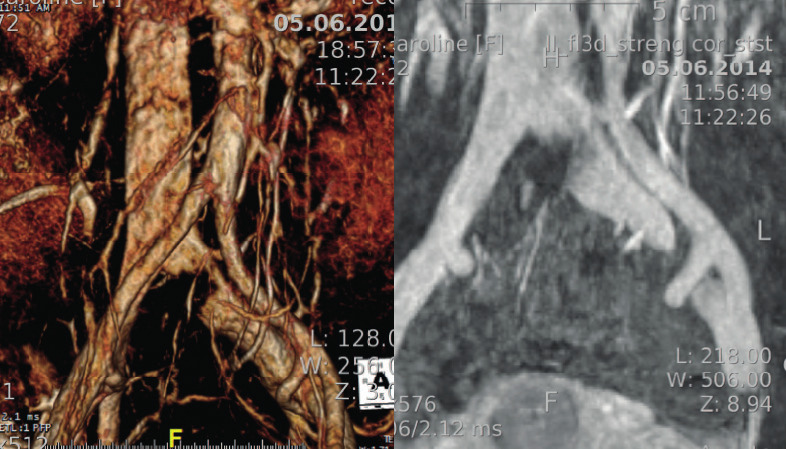
— Confirmation by MIR & Venography.

### Interventions

All patients signed informed consent forms prior to surgery and provided written informed consent for the use and publication of case details, personal information, images, and videos, including their faces. Pre and post interventional pain was assessed by visual analogue scale (VAS score).

No ethical approval was needed for this retrospective study, since the interventions were based on a routine clinical practice for referred patients in the Possover Medical Center.

The decision to carry out an endovascular intervention (embolization, angioplasty, stenting) was made by radiologists. An endovascular treatment was considered successful if the reduction is VAS score was more than 50% at 6 months follow-up. Embolization of the ovarian veins as in a Pelvic Congestion Syndrome is not a therapeutic option of MTS or even increases the downstream venous pressure and thus may aggravate the symptomatology of the patient.

If the pain reduction was less than 50%, a laparoscopic exploration and decompression of the pelvic somatic nerves suspected to be involved in pain generation was performed (Table 3) (Possover, 2011;Possover, 2010).

**Table III:** Characteristics of the 47 patients suffering from “Pelvic Somatic Pain”.

Sacral Radiculopathy L4-L5-S1 (supralevator portion of the sacral plexus)		n=23
Sacral Radiculopathy S2-S4 (infralevator portion of the sacral plexus)		n=9
Isolated Pudendal Pain (endopelvic portion of the pudendal nerve)		n=15
Numeric pain perception scores (VAS)		8.1 (±1.6; 4-10)
Urinary urgency:		
	- Bladder hypersensitivity	n=42
	- Bladder hyperactivity (iOAB)	n=2
Lateral parametric veins at trigger point (by vaginal sonography):		
	- Diameter	8.6mm (±1.8; 6-11)
	- Reverse flow (reflux)	n=47
Post-thrombotic stigmata:		
	- Pigmentation changes in the lower extremities	n=2
	- Varicose veins in the lower extremities (left>right)	n=39
	- Phlebitis or skin ulcers	n=1 (phlebitis)
	- Pelvic sidewall phleboliths (by vaginal palpation)	n=17

Laparoscopic access to the pelvic somatic nerves is obtained by:

- Dissection of the pararectal space and transection of the sacral hypogastric fascia to expose the infra- cardinal part of the sacral plexus (S#1-4/5).
- Dissection of the lumbosacral fossa outside the external iliac vessels and mobilization of the interiliac nodes from the pelvic wall for exposure of the supracardinal part of the sacral plexus (L#5, S#1-4/5), the endopelvic portion of the sciatic nerve and of the pudendal nerve (Fig. 4).

**Figure 3:**
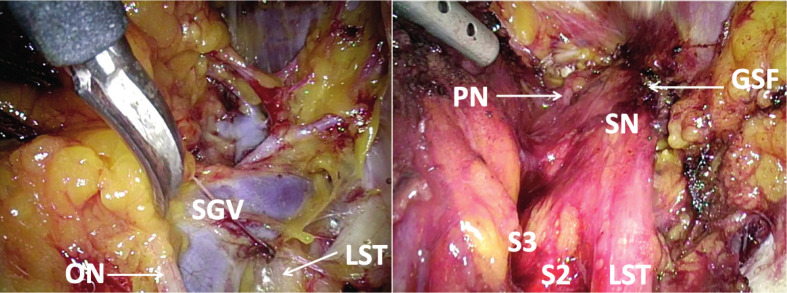
— Laparoscopic exploration/decompression of the sacral plexus by an enlarge and atypical superior gluteal vein (Left: before the decompression – Right: after the decompression). ON: obturator nerve – SGV: superior gluteal vein – LST: lumbosacral trunk – PN: pudendal nerve – SN: sciatic nerve – GSF: greater sciatic foramen – S: sacral nerve root.

Following laparoscopic nerve decompression, all patients received Pregabalin 75mg x2/d (if this therapy hadn’t already been started before the intervention) and we advised the patients to increase the doses by steps of 50mg per week depending on pain intensity, under control of their general practitioner.

The evolution of the pain was recorded on a monthly basis over a period of one year recorded during post-operative consultations or by e-mail for patients living abroad.

All statistical analyses were conducted by using SPSS/PC for Windows (SPSS Inc., Chicago, IL, USA). Data are presented as mean -standard deviation (SD). For all analyses, the level of statistical significance was set at P less than 0.01 (paired t-test SPSS).

## Results

### 

The clinical flow chart is shown on Table 2. Sixty-one consecutive patients were included in this clinical study. Mean age was 34 years (23y- 51y). Forty-two patients were nulliparous. Fourteen patients presented with visceral pain, forty-seven with somatic neuropathic pelvic pain.

**Table I:**
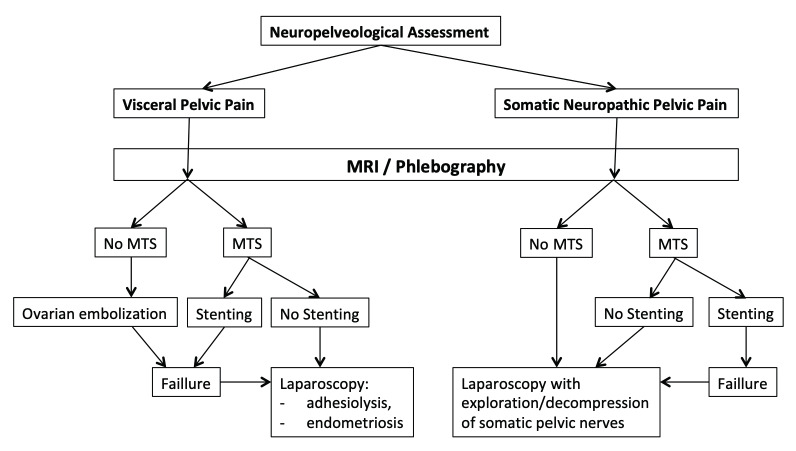
— Decision flow chart.

### Patients with Visceral pelvic pain (n=14)

Eleven patients underwent unilateral embolization of the left ovarian vein, and two had bilateral embolization of the ovarian veins. The successful embolization rate was 100% with no significant complications during or after embolization. An improvement of pain was observed in all patients - pain decreased by 3.93 points on average, from 7.21 (±1.42; 4-10) to 3.28 pts (±1.54; 1-6) in 6 months follow-up (p<0.01). In 4 patients, due to an insufficient pain improvement, laparoscopy was performed with intraoperative detection of small peritoneal endometriosis spots or adhesions. Pain significantly improved in these four patients.

In one patient, due to the development of a non-neurogenic left genitofemoral neuropathy approximately 9 months after embolization, laparoscopy was indicated: Coils of the left ovarian vein were found in direct contact with the genitofemoral nerve at the anterior surface of the psoas muscle. The vein was resected and the coils removed. The neuropathic pain almost disappeared during the months following the procedure.

In one patient, MRI/Phlebograpy showed MTS, which required endosvascular angioplasty. At 6 months follow-up, due to the persistence of pain symptoms, laparoscopy was performed: some adhesions and small spots of peritoneal endometriosis were found and treated, with significant pain relief.

### Patients with Somatic pelvic pain (n=47)

Some of the patients included in the group “Somatic pelvic pain” group were also suffering from pelvic visceral pain, but predominant symptoms were the somatic pains.

MRI showed MTS requiring endovascular stenting in nineteen patients. Despite a degree of initial pain relief in some patients, there was no consistent pain relief at 6 months follow-up in any of the patients undergoing stenting for MTS (VAS reduction <10%). In the remaining 28 patients imaging found minimal MTS or NCS which did not require either an angioplasty or a stenting.

All forty-seven patients underwent laparoscopic exploration with decompression of the left pelvic nerves in forty-one patients, bilateral in six patients.

Regardless of whether the preoperative assessment was suggestive of entrapment of sciatic L5-S1, sacral radioculopathy S2-S4 or neuralgia of the endopelvic portion of the pudendal nerve, the laparoscopic intervention consisted of the systematic exploration of all three anatomical regions with coagulation/transection of all veins showing a direct contact with the nerves (Possover M, Forman A, 2015). All veins compressing the nerves were transected after meticulous bipolar coagulation; neither ligatures nor clip were used.

No major intraoperative complications occurred and no conversion to open surgery was required. All patients reported some slight weakness in the leg (especially in leg adduction) due to manipulation of the obturator nerve for access to the sciatic nerve) and some hypoesthesia especially in genital areas, which subsided spontaneously after several days or weeks in all patients. One patient presented with a pelvic hemorrhage two months after the intervention following a vaginal examination extra muros of the operated area. Since then, we advise our patients to strictly avoid any digital manipulation/massage of the pelvic sidewall for at least four months after such a neuropelveologic procedure.

**Figure 5:**
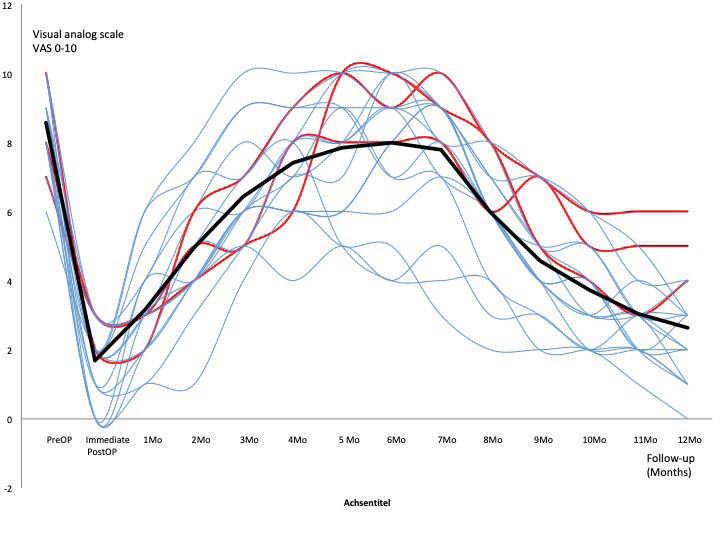
— volution of VAS of patients after LSC nerves decompression in patients secondary to endovascular intervention by significant MTS (n=19). Blue lines: patients with pain decreased >50% (n=16) Red lines: patients with pain decreased 30-50% (n=3) Black line: mean value.

**Figure 6:**
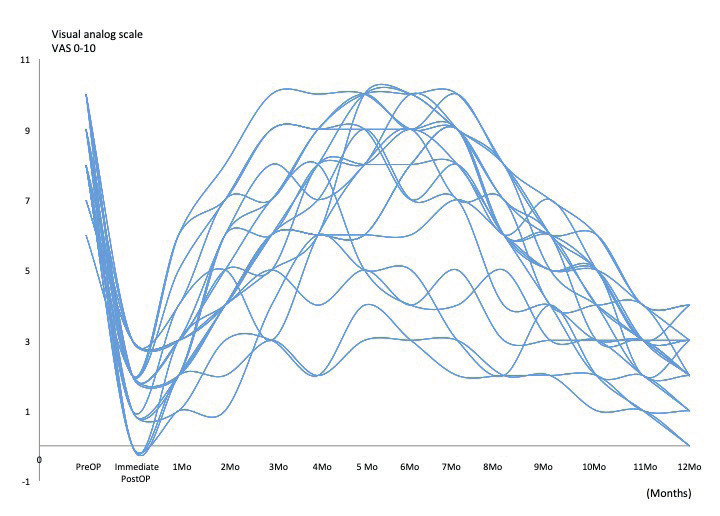
— Evolution of patients who did not required any endovascular treatment and with a VAS reduction >50% after LSC nerves decompression (n=29).

**Figure 7:**
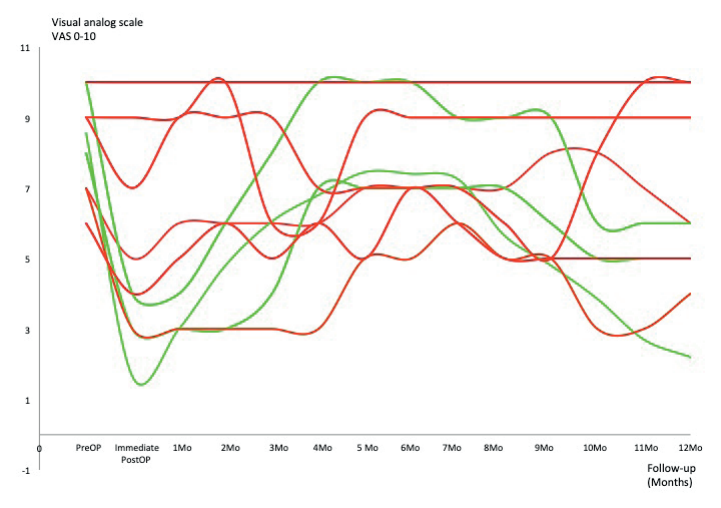
— Evolution of patients who did not require any endovascular treatment and with a VAS reduction 30-50% (green lines – n=3) or <10% (red lines – n=5) after LSC nerves decompression.

In the nineteen patients who had an endovascular treatment for MTS, we found intraoperative dilated veins >1cm in diameter (especially the gluteal and pudendal veins) but also “caput medusa” formations covering and entrapping the sciatic nerve, the sacral roots and/or the endopelvic portion of the pudendal nerve. Sixteen of them reported at 1-year-follow-up a pain reduction ≥50% (84.2%) and three a pain reduction 30-50% (15.8%) – with an overall VAS reduction from 8.56 (±1.17;7-10) to 2.63 (±1.53; 0-6) at one-year- follow-up (p<0.01). Figure 5 shows pain evolution: after a significant pain relief of several days after the procedure (due to the intervention-induced neurapraxia), pain reappeared and increased over a period of 6-8 months after the procedure before the pain started slowly to decrease to a more or less constant level about 12 months after the procedure. In the further 28 patients who did not require an endovascular intervention before their laparoscopy, twenty (71.4%) experienced a pain reduction ≥50% (Fig. 6), three (10.7%) a pain reduction 30-50% and five (17.85%) had no significant improvement (Fig. 7) – overall VAS decreased from 8.4 (±1.23; 6-10) to 3.53 (±2.67; 0-10) at one-year follow-up.

The long-term pain evolution seemed to be correlated with pain improvement right after the procedure: Those patients who had the least pain immediately after the operation, had the best results at one year. On the contrary, patients who did not feel any difference in the intensity of pain in the days following the procedure, did not benefit from the procedure at 12-months follow-up.

## Discussion

Chronic Pelvic Pain is a major challenge to healthcare providers because of their unclear etiology, complex natural history and poor response to therapy. Pathology of the pelvic nerves and plexuses may explain such “unknown pain conditions” and associated pelvic organs dysfunctions. However, the current understanding of pelvic nerve pathology is generally limited to pathology of the spinal cord with herniated disc responsible for sciatic pain, and the Alcock’s canal Syndrome responsible for genital pain when sitting. However, pathologies of the pelvic nerves may explain many cases of chronic pelvic pain syndrome including neuropathic pain in the lower back, the genito-anal areas and the lower extremities, but also pelvic organ dysfunctions. The incidence of pelvic nerve pathologies seems widely underestimated, mainly due to a lack of awareness that such lesions may exist, a lack of diagnosis and acceptance, as well as declaration and report of such lesions. Considering the number of pelvic pathologies, pelvic tumors, endometriosis of the pelvic nerves and invasive procedures in proximity of the pelvic nerves that could potentially induce neuronal compression, entrapment or damage, reports in the literature are rare.

Pelvic congestion syndrome, also known as pelvic vein incompetence, is also a condition responsible for Chronic Pelvic Pain due to enlarged pelvic veins in the lower abdomen. The fact that pelvic dilated veins may also induce pelvic somatic neuropathic pain is much less known. Even less known in Gynecology are two different pathologies of the venous system responsible for the formation of pelvic varicose veins predominant on the left, the MTS and the NCS. While MTS was initially presumed to be rare when it was first anatomically defined in 1957 (Kibbe MR, Ujiki M et al, 2004), the population burden of this condition is unknown, and it may be higher than generally perceived (García-Gimeno M, Rodríguez-Camarero S et al, 2009). In our series, no patient had the suspicion of an MTS or a Pelvic Congestion Syndrome mentioned before. The patients did not present any history of vascular diseases and clinical examination did not reveal any vascular abnormalities except the presence of predominant varicose veins of the left leg in 39 of them, and pelvic sidewall/paravaginal phlebolyte on vaginal examination (n=17). The only reason that led to evoke an MTS/NCS was uterine varicose veins predominant on the left side, visualized by vaginal ultrasonography with Doppler examination of the uterine vessels. In the management algorithm of pelvic venous disorders, Doppler examination is the first-line imaging investigation. Doppler cannot identify all reflux, but it is a very good tool for identifying pelvic varicose veins. It allows to consider the main obstructive and supplying syndromes. In those cases, diagnosis will be confirmed by a second-line, cross-sectional imaging examination (angioMR, angioCT). Vaginal ultrasonography with Doppler of the uterine vessels is within the reach of most gynecologist, is easy to perform and discovery of an unexpected MTS or NCS may be life saving as this condition increases the risk of deep veins thrombosis and pulmonary embolism (Greiner M, 2005). This is even more of importance in Gynecology since hormone-based treatments are widely considered even as first line-therapy for visceral pelvic pain, while they increase the risk of thrombosis and embolism. The selective retrograde pelvic venography is the only imaging technique that is able to achieve an accurate anatomic and hemodynamic mapping of the pelvic varicose veins, but it must remain a pre-treatment investigation. Because these patients are at elevated risk of developing an extensive left iliofemoral deep vein thrombosis, the first line treatment may be the treatment of the vascular stenosis, even if - according to our results - endovascular intervention may not decrease the volume of the pelvic sidewall varicose and therefore may not improve pain significantly in patients with pelvic somatic neuropathies. We also should consider that pelvic veins are not independent but interconnected as a network and connected with other networks, particularly with the lower-limb veins. This connectivity explains why an abdominal or pelvic venous reflux can be the origin of a venous anomaly located in another area, ie, a left ovarian reflux can supply the right perineal varices and may induce pain such as vulvodynia or even coccygodynia (Greiner M, Faye N, 2012). In other words, the absence of pelvic congestion syndrome is not a sufficient argument for not treating the pelvic varicose. The aim of the laparoscopic exploration is to release the pelvic somatic nerves from all enlarged veins showing direct contact to them, but not all pelvic dilated veins must be removed. Indeed, MTS can also cause development of extensive retroperitoneal and pudendal venous collaterals, which shunt the distal iliofemoral venous system to the contralateral deep venous vessels. Both of these conditions create alternative venous drainage to counteract the venous stasis, preventing overwhelming venous obstruction. Although they can be unsightly, these conditions have an important hemodynamic function and should not be removed for cosmetic or preventive reasons.

In view of our results, it is important to inform patients after laparoscopic nerves exploration/ decompression, that after a pain improvement of several days/weeks, the pain reappears in most patients (>95% of the patients according to our experience over the last 15 years) and even increases. Pain relief may take up to 8 months after the procedure so it makes no sense to reduce pain therapy before. Further pain, tingling sensations, electrical shots may appear in sacral/pudendal dermatomes. In patients who do not appear to experience any improvement in pain in the days following the laparoscopic nerves decompression, the long-term results are not good enough and treatment has failed.

Our study has the major limitation to be a retrospective study. These drawbacks can be overcome by prospective, randomized, and controlled trials with a larger number of participants.
